# Good vibes keep worries at bay: a prefrontal-hypothalamic pathway engages positive affect to mitigate fear

**DOI:** 10.1172/JCI211427

**Published:** 2026-10-15

**Authors:** Quan-Son Eric Le, Luis E. Rosas-Vidal

**Affiliations:** Stephen M. Stahl Center for Psychiatric Neuroscience, Department of Psychiatry and Behavioral Sciences, Northwestern University Feinberg School of Medicine, Chicago, Illinois, USA.

## Abstract

Trauma-related neuropsychiatric disorders are commonly treated with exposure-based therapy, an approach based on fear extinction, in which fear memory and expression are suppressed by repeated exposure to the inciting fear stimulus. Fear extinction–focused research often investigates mechanisms of fear inhibition, but positive affect may also contribute to facilitating extinction. In this issue of the JCI, Lin et al. used activity-dependent neuronal labeling in a model of fear extinction to tag extinction-activated medial prefrontal cortex (mPFC) ensembles, identifying functionally distinct projections to the zona incerta (ZI) and supramamillary nucleus (SuM). While mPFC-ZI projections governed extinction retrieval, mPFC-SuM projections encoded positive affect and reinforced fear extinction persistence. Additionally, Lin et al. discovered that SIRT1-expressing mPFC-SuM extinction-recruited projections control extinction relapse vulnerability, showing that SIRT1 overexpression can be leveraged to reduce fear relapse. Overall, this study shows that key cortico-hypothalamic projections manage extinction retrieval and positive affect to promote fear extinction.

## Positive affective processes may contribute to extinction memory

Fear extinction is the neurobiological underpinning of exposure-based therapies used to treat trauma-related neuropsychiatric disorders. In the context of therapy and in experimental settings, fear extinction involves suppressing a learned fear memory and its associated fear expression (1, 2) by repeatedly presenting the learned fear stimulus in the absence of a paired aversive stimulus. However, fear extinction does not erase the original fear association, and thus fear relapses can occur, interfering with the effectiveness of extinction-based therapy for trauma-related disorders (3, 4). Thus, it has been a longstanding goal to improve clinical outcomes by optimizing key aspects of fear extinction, including its acquisition, its resistance to fear relapse, and its ability to mitigate strong fear reactions that are evoked by the original fear memory in the early phases of exposure. Importantly, these features of fear extinction should occur without impairing important underlying memory processes.

Extinction substantially engages the medial prefrontal cortex (mPFC), which governs adaptive behaviors and affective processing (5, 6) and contains heterogeneous neuronal populations that exert top-down control of behaviors and cognition (7–10). While specific extinction-recruited mPFC ensembles have been identified (11), it is unclear if these ensembles display functional heterogeneity for extinction retention and positive affect. In this issue, Lin et al. set out to uncover the functional architecture of mPFC extinction-recruited ensembles and whether the ensembles contain neurons encoding positive affect (12).

## Distinct mPFC subpopulations engage extinction and positive affect

Lin et al. studied fear extinction in mice trained to associate an auditory cue with an incoming electrical foot shock. This conditioning regimen leads to freezing behavior when the cue is presented. Extinction training involved repeatedly presenting the cue in the absence of foot shock, and fear extinction was measured by the reduction in freezing (Figure 1). To find the neuronal populations within the mPFC involved in fear extinction encoding, Lin et al. combined ensemble capture strategies that label extinction-activated neurons with fear extinction behavioral tests to identify mPFC neurons that were activated during the extinction session (mPFC^Ext^ neurons). Use of optogenetics to selectively target this identified ensemble of neurons revealed that the mPFC^Ext^ neuronal ensemble mediated both extinction performance and positive affect (12). Lastly, the authors further confirmed that the mPFC ensembles show heterogeneous firing patterns during extinction retrieval- and positive affect-inducing experiences (12). Thus, the authors identified a subset of mPFC^Ext^ neurons that encoded positive affect rather than extinction retrieval, showing that positive affect contributes to extinction. This finding raises the question of whether extinction learning is rewarding or whether positive affect mPFC^Ext^ neurons are repurposed to construct an extinction memory.

## mPFC-SuM and mPFC-ZI projections govern extinction and affect

Using anatomical tracing, the authors found that mPFC^Ext^ neurons were glutamatergic and mostly pyramidal neurons (12), which projected to multiple regions involved in emotional processing or conditioned reward (13–15). The authors then targeted each mPFC projection to measure its activity during extinction retrieval or water intake. Fiber photometry and single-unit recordings revealed that mPFC^Ext^ projections to the supramammilary nucleus (SuM) were active during positive affective experiences, while mPFC^Ext^ projections to the zona incerta (ZI) were active during extinction retrieval (ref. 12; Figure 1). Lastly, sparse labeling was used to show that mPFC^Ext^-ZI projecting neurons were morphologically different from mPFC^Ext^-SuM projecting neurons (12), further cementing that these two mPFC projections are distinctly separate in function, anatomy, and morphology.

## mPFC-ZI projections support extinction retrieval via somatostatin-expressing neurons

To further detail the architecture of downstream mPFC^Ext^ targets, RNAscope was used to analyze subpopulations of GABAergic neurons in the ZI and found that somatostatin-expressing (SST-expressing) neurons were the dominant target of PFC^Ext^ projections (12). Additional optogenetic and fiber photometry testing revealed that exciting mPFC^Ext^-ZI terminals increased activity in ZI^SST^ neurons, and inhibiting mPFC^Ext^-ZI^SST^ projections suppressed extinction retrieval but not positive affect, as assessed by sucrose preference (12). Optogenetically exciting mPFC^Ext^-ZI projections facilitated extinction expression without influencing positive affect (12). Thus, these findings demonstrate that the mPFC^Ext^-ZI^SST^ projection is a dedicated circuit for extinction expression absent of positive affective processes.

## mPFC-SuM projections modulate positive affect and extinction persistence

Similar methods were utilized to identify and investigate glutamatergic neurons in the SuM downstream of the mPFC. Inhibition of mPFC^Ext^-SuM projections disrupted sucrose preference without affecting extinction retrieval, and manipulating SuM^Glu^ neurons controlled the return of fear response during spontaneous recovery tests (12), showing that they affect both long-term extinction persistence and positive affect. Finally, activation of these neurons reduced long-term fear responses and increased place preference (12), indicating that the mPFC^Ext^-SuM^Glu^ pathway manages positive affect and is required for long-term extinction persistence.

## Extinction persistence relies on mPFC-SuM SIRT1 expression

Single-nucleus RNA-seq was then used to define the molecular signature of the SuM-projecting mPFC^Ext^ ensemble, finding that this ensemble was enriched for SIRT1 (12), a protein pivotal for managing pain vulnerability and antidepressant effects (16, 17). Furthermore, offline enhancement of neuronal activity of this same ensemble led to enhanced persistence of extinction (resistance to spontaneous recovery) and an associated increase in SIRT1 expression (12).

Single prolonged stress (SPS) leads to the development of a post traumatic stress disorder–like (PTSD-like) phenotype that recapitulates resistance to fear extinction and enhanced spontaneous recovery of fear (18). Using this SPS model in combination with a conditional CRISPR-Ca9 strategy to enable knockout of SIRT1 from SuM-projecting mPFC^Ext^ ensembles, the authors observed enhanced freezing during the spontaneous recovery test. Repeating the SPS model with ensemble-specific overexpression of SIRT1 blocked spontaneous recovery of fear. Together, these results demonstrate that SIRT1 acts within the SuM-projecting mPFC^Ext^ ensemble to improve extinction persistence and mitigate spontaneous recovery toward threat-reminding stimuli even when extinction mechanisms are deficient. These results collectively showcase a mechanism in which positive affect contributes to long-term extinction persistence and points to various potential strategies to improve extinction learning.

## Limitations and potential future directions

The results point to various possibilities to improve extinction learning and clinical outcomes of exposure therapy. These include testing if administration of SIRT1 agonists like resveratrol after therapy may prevent future relapse of symptoms. Another possibility could be testing whether directly targeting positive affect behaviorally or pharmacologically during extinction could lead to enhanced recruitment of mPFC^Ext^-SuM neurons and resistance to fear relapse.

Chronic pain is frequently comorbid with PTSD (19). Future studies should investigate whether chronic pain and persistent PTSD symptoms are characterized by shared neurobiological disruptions involving the mPFC-SuM pathway or alterations in SIRT1 expression. This line of research may be particularly valuable because it could help identify clinical biomarkers, improve understanding of the mechanisms underlying chronic pain–PTSD comorbidity, and reveal therapeutic targets to improve treatment of these two conditions. Furthermore, an additional question that could stem from this line of research could be whether deficient processes related to positive affect are a risk factor for developing chronic pain and/or PTSD.

One of the most intriguing findings in the current study involved allowing water-deprived mice to consume water, a reliable method of evoking positive affect. A subset of mice consumed significantly less water, which could be interpreted as reduced sensitivity to the positive affective experience. The mice with low water intake displayed greater freezing during spontaneous recovery tests, suggesting that they are more vulnerable to regaining the fear response. However, the study measured water intake only after fear conditioning and extinction training. The lack of information about preconditioning water intake raises the question of whether low-intake mice had a preexisting affective deficit or experienced anhedonia after fear conditioning due to another underlying vulnerability to traumatic stress.

Activation of the mPFC^Ext^-SuM pathway had a greater effect in suppressing spontaneous recovery in low-water intake mice compared with its effect in the normal-intake population. This may reflect that augmenting the positive affective mPFC^Ext^-SuM pathway suppresses fear expression in this vulnerable group. However, despite activation of the mPFC^Ext^-SuM pathway, low-water intake mice displayed 40% freezing during the spontaneous recovery test compared with approximately 30% freezing in the normal-intake mice, suggesting that stimulating this projection may not be sufficient to fully suppress fear expression in this more vulnerable subgroup. Achieving freezing levels closer to those of the normal-intake group may require more robust manipulations or concurrent engagement of additional pathways. For example, simultaneous activation of mPFC^Ext^-SuM with the mPFC-ZI projection may provide synergistic effects for full suppression of fear responses.

While Lin et al. demonstrated that the mPFC^Ext^ ensemble projected onto glutamatergic SuM neurons, as well as showcased a role for mPFC^Ext^-SuM^SIRT1^ projections, they did not perform verification of Glu/SIRT1 overlap within these populations. There still is a possibility that only a subset of glutamatergic SuM neurons are also SIRT1-positive; thus, this subset and other SIRT1-expressing cell types may also contribute to extinction persistence in the context of SIRT1 overexpression.

Overall, the findings produced within this article substantially contribute to the field of affective neuroscience and provide a mechanistic link between valence and motivated behaviors as a key factor in improving extinction persistence. Future studies could use the same methods to explore downstream projections of the central nucleus of the amygdala, another region that governs valence-based responses, reward processing, and fear extinction (20). Additionally, the findings imply that therapeutic treatments could employ personalized strategies that explicitly activate and enhance positive affective pathways to improve fear extinction in trauma-related disorders.

## Conflict of interest

The authors have declared that no conflict of interest exists.

## Funding support

- LERV is supported by a NARSAD Young Investigator Grant from the Brain & Behavior Research Foundation.

## Figures and Tables

**Figure 1:**
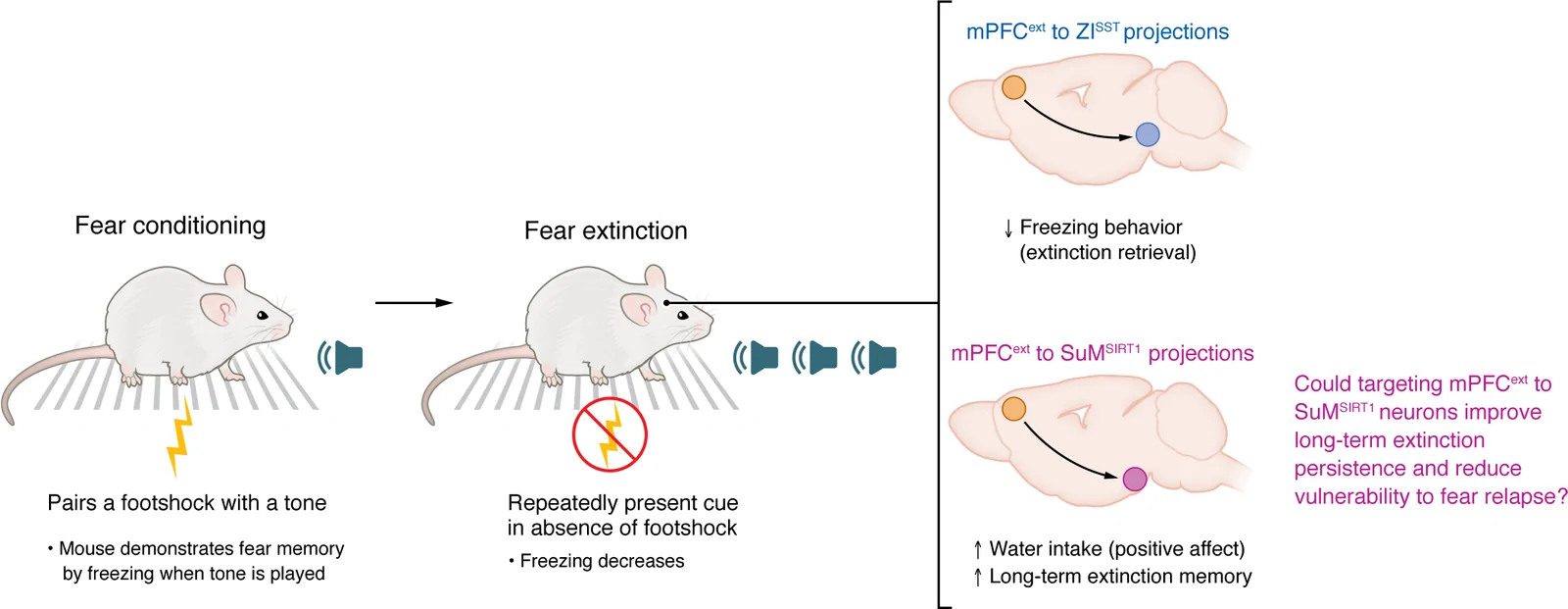
Distinct mPFC projections encode fear extinction retention and positive affect. In a mouse model of fear extinction, Lin et al. (12) identified two ensembles of mPFC neurons engaged by extinction training (mPFC^ext^) and used optogenetic inhibition and activation of these projections to tease out their roles in behavioral expression. Extinction retrieval was assessed by reduction in freezing behavior to a conditioned stimulus (tone), and positive affect was assessed using water intake. An mPFC-to-ZI^SST^ ensemble projected to SST-expressing neurons of the zona incerta; its activation was required for extinction retrieval but did not affect positive affective processes. In contrast, an mPFC-to-SuM^SIRT1^ ensemble projected to SIRT1-expressing neurons of the supramamillary nucleus and encoded both positive affect and long-term persistence of extinction retrieval, which Lin et al. interpreted as controlling vulnerability to fear relapse. The mPFC-to-SuM^SIRT1^ projection could therefore represent a target for suppressing fear expression in PTSD and other trauma-related neuropsychiatric disorders.

## Version 1 10/15/2026

Electronic publication
